# Salvianolic Acid B Ameliorates Lipopolysaccharide-Induced Albumin Leakage from Rat Mesenteric Venules through Src-Regulated Transcelluar Pathway and Paracellular Pathway

**DOI:** 10.1371/journal.pone.0126640

**Published:** 2015-05-19

**Authors:** Chun-Shui Pan, Ying-Hua Liu, Yu-Ying Liu, Yu Zhang, Ke He, Xiao-Yuan Yang, Bai-He Hu, Xin Chang, Ming-Xia Wang, Xiao-Hong Wei, Jing-Yu Fan, Xin-Min Wu, Jing-Yan Han

**Affiliations:** 1 Tasly Microcirculation Research Center, Peking University Health Science Center, Beijing, China; 2 Key Laboratory of Carcinogenesis and Translational Research (Ministry of Education), Peking University Cancer Hospital& Institute, Beijing, China; 3 Department of Integration of Traditional Chinese and Western Medicine, School of Basic Medical Sciences, Peking University, Beijing, China; 4 Key Laboratory of Microcirculation, State Administration of Traditional Chinese Medicine of the People's Republic of China, Beijing, China; 5 Key Laboratory of Stasis and Phlegm, State Administration of Traditional Chinese Medicine of the People's Republic of China, Beijing, China; University of Illinois at Chicago, UNITED STATES

## Abstract

Lipopolysaccharide (LPS) causes microvascular barrier disruption, leading to albumin leakage from microvessels resulting in a range of disastrous sequels. Salvianolic acid B (SalB) is a major water-soluble component derived from Salvia miltiorrhiza. Previous studies showed its potential to attenuate microvascular barrier dysfunction, but the underlying mechanism is not fully understood. The present study was intended to investigate the impact of SalB on endothelial cell barrier in vivo in rat mesenteric venules as well as in vitro in human umbilical vein endothelial cells (HUVECs), aiming at disclosing the mechanism thereof, particularly the role of Src in its action. Male Wistar rats were challenged by infusion of LPS (2 mg/kg/h) through left femoral vein for 90 min. SalB (5 mg/kg/h) was administrated either simultaneously with LPS or 30 min after LPS infusion through the left jugular vein. Vesicles in venular walls were observed by electron microscopy. HUVECs were incubated with LPS with or without SalB. The expression of Zonula occluden-1 (ZO-1), VE-cadherin, caveolin-1 and Src in HUVECs was assessed by Western blot and confocal microscopy, binding of SalB to Src was measured using Surface Plasmon Resonance and BioLayer Interferometry. Treatment with SalB inhibited albumin leakage from rat mesenteric venules and inhibited the increase of vesicle number in venular endothelial cells induced by LPS. In addition, SalB inhibited the degradation of ZO-1, the phosphorylation and redistribution of VE-cadherin, the expression and phosphorylation of caveolin-1, and phosphoirylation of Src in HUVECs exposed to LPS. Furthermore, SalB was found able to bind to Src. This study demonstrates that protection of SalB against microvascular barrier disruption is a process involving both para- and trans-endothelial cell pathway, and highly suggests Src as the key enzyme for SalB to work.

## Introduction

Lipopolysaccharide (LPS) is an essential component of the cell wall of Gram negative bacteria, which causes a series of inflammatory reactions in the infected subjects, including microvascular barrier disruption. LPS-induced microvascular barrier disruption allows for infiltration of leukocytes to the infection site, but also leads to plasma albumin flux from blood stream to interstice. Albumin leakage from microvessels represents a critical episode in the pathogenesis of a wide variety of clinical conditions, such as endotoxmia, adult respiratory distress syndrome and shock [1, 2]. However, the strategy directed at preserving microvescular barrier remains limited in clinic so far.

The microvascular barrier is maintained by the integrity of microvascular endothelium and regulated by transcellular pathway and paracellular pathway[3]. Transcellular pathway is widely accepted as a caveolae-mediated vesicular transport across endothelial cells. Caveolae are lipid rafts in plasma membrane with diverse proteins, and act as a signaling platform during inflammation [4], with caveolin-1 being the primary structural component and functional regulator. Paracellular pathway is controlled by endothelial cell-cell junctions [5], including tight junctions (TJs) and adherens junctions (AJs). Claudins, occludin, and junctional adhesion molecules (JAMs) are the major transmembrane proteins localized at TJs, while zona occludins (ZO), the intracellular components of TJs associate with actin cytoskeleton to stabilize TJs. AJs are composed of vascular endothelial cadherin (VE-cadherin) as the principal protein with catenins linking to actin cytoskeleton [6]. Among the junction proteins identified, occludin, claudin-5, JAM-1, ZO-1 and VE-cadherin are known to play a vital role in microvascular barrier dysfunction induced by LPS and pro-inflammatory mediators [7–9]. Several mediators and signing pathways have been reported to be implicated in regulation of microvascular permeability, of which Src family protein tyrosine kinases received increasing attention in recent years [3]. LPS recognition by TLR4 activates the c-Src [10], which in turn phosphorylates caveolin-1 at tyrosine 14, promotes caveolae to shuttle across endothelial cells and transport plasma albumin [11, 12]. In addition, Src phosphorylation promotes NF-κB nuclear translocation and increases caveolin-1expression [13]. Src is also reported to increase the phosphorylation of VE-cadherin [14]. The facts described above indicate that Src is the key molecule that regulates both transcellular pathway and paracellular pathway [15]. However, no strategy is available as yet in clinic to prevent or attenuate LPS-induced microvascular barrier dysfunction by acting on Src.

Salvianolic acid B (SalB), is a major water-soluble component derived from Salvia miltiorrhiza with a phenolic hydroxyl group. Salvia miltiorrhiza, also known as Danshen, is a herb medicine which is used, either alone or in combination with others, for prevention and treatment of cardio- and cerebral vascular diseases, such as angina pectoris, hyperlipidemia, and acute ischemic stroke [16]. Accumulating study has been published to explore the mechanism and target organs of Danshen and its active compounds [17]. SalB has been reported to possess multiple desirable potentials, including anti oxidative activity, ability to reduce leukocyte-endothelial adherence, inhibition of inflammation and metalloproteinases expression and indirect regulation of immune function [18]. Studies in our laboratory over the past years suggest that SalB is able to protect microvascular barrier dysfunction [19–22]. However, the mechanism thereby SalB attenuates microvascular hyperpermeability is unclear at present. We hypothesized that SalB preserves microvascular endothelium barrier via interference in Src activation. To test this hypothesis, in the present study, we assessed the effect of SalB on LPS-elicited rat mesenteric venular hyperpermeability, and explored the underlying mechanism both in vivo in rat and in vitro in HUVECs with particularly focusing on the role of Src.

## Materials and Methods

### Reagents

LPS was purchased from Sigma (St. Louis, Missouri, USA). SalB was purchased from the National Institute for the Control of Pharmaceutical and Biological Products (Beijing, China). Cell culture plastic wares were from Costar (Cambridge, Massachusetts, USA). BIAcore 3000 CM5 series sensor chips were from BIAcoreAB (Piscataway, New Jersey, USA). All other chemicals used were of the highest grade available commercially.

### Animals

Male Sprague-Dawley rats (220 ± 20 g) were provided by the Animal Center of Peking University Health Science Center (Beijing, certificate no. SCXK 2006–0008). The rats were fasted for 12 h before experiment, while allowing for free access to water. The experimental procedures were carried out in accordance with the European commission guidelines (2010/63/EU). All animals were handled according to the guidelines of the Peking University Animal Research Committee. The protocols were approved by the Committee on the Ethics of Animal Experiments of the Health Science Center of Peking University (LA2011-38).

### Study design

The rats were randomly divided into four groups; the animal numbers for assessment of each variable in different groups are detailed in Table 1. All animals underwent 10 min basal observation, and afterwards, were cannulated with 2 catheters, one through the left jugular vein for infusion of SalB solution, and the other through left femoral vein for infusion of LPS, whenever needed. The dose of LPS and SalB infused was 2 mg/kg/h and 5 mg/kg/h, respectively. In LPS group LPS solution in saline was infused for 90 min via left femoral vein. In SalB + LPS group, SalB and LPS solution were infused through left jugular vein and left femoral vein simultaneously for 90 min. In LPS + SalB group, SalB solution was infused through left jugular vein starting from 30 min after LPS administration. The same volume of saline was infused in NS group for 90 min via left femoral vein.

**Table 1:** The number of animals for different experimental groups and various parameters.

	NS	LPS	SalB+LPS	LPS+SalB	Total
Albumin leakage	6	6	6	6	24
Ultrastructure	3	3	3	3	12
Total	9	9	9	9	36

NS: normal saline group, animals received normal saline via both left jugular vein and left femoral vein for 90 min; LPS: LPS group, animals received normal saline via left jugular vein and LPS (5 mg/kg/h) via left femoral vein for 90 min; SalB+LPS: animals received 2 mg/kg/h SalB through the left jugular vein and, concomitantly, 5 mg/kg/h LPS through the left femoral vein, for 90 min; LPS+SalB: animals received 5 mg/kg/h LPS through the left femoral vein for 90 min, and 2 mg/kg/h SalB through the left jugular vein 30 min after the initiation of LPS infusion.

### Determination of albumin leakage from mesentery venules

To quantify albumin leakage across mesenteric venular walls, the animals were intravenously injected with 50 mg/kg of FITC-labeled bovine serum albumin (Sigma Chemical, St. Louis, Missouri, USA) 10 min before each experiment. Fluorescence signal (excitation wave length at 420 to 490 nm, emission wave length at 520 nm) was acquired using a silicon-intensified target camera (C-2400-08; Hamamatsu Photonics, Hamamatsu, Japan). Albumin leakage was estimated by dividing the fluorescent intensity in the perivenular interstitium (Ip) by in the venules (Iv), and the dynamic alteration of albumin leakage was expressed as the ratio of albumin leakage at different time points to that of the baseline.

### Ultrastructure examination

After experiment, rats underwent perfusion through the left ventricle with physiological saline followed by 120 mL of phosphate-buffered 4% (w/v) paraformaldehyde plus 2% (w/v) glutaraldehyde at a speed of 3 mL/min. The mesentery tissues were removed and further fixed by immersion in phosphate-buffered 3% (w/v) glutaraldehyde for 1 h. The tissues were routinely processed for transmission electron microscopy and examined in JEM 1230 (JEOL, Tokyo, Japan).

### Cell culture and treatment

HUVECs, purchased from ATCC (ATCC Number: PCS-100-013, VA, USA) were cultured in ECM medium containing 10% FBS, ECGS, 100 U/ml of penicillin and streptomycin (ScienCell Research Laboratories San Diego, California, USA) at 37°C in a 5% CO2–95% air environment. HUVECs were passaged by using 0.25% trypsin, and passages 5–6 were used in all experiments. The medium was changed every other day until the cells became confluent.

After serum-free culture for 18 h, HUVECs were divided into 6 groups (n = 6) as follows: (1) SalB group: cells were treated with SalB (10^–6^ M) for 90 min; (2) LPS 30 group: cells were treated with 100 ng/mL LPS for 30 min; (3) LPS 90 group: cells were treated with 100 ng/mL LPS for 90 min; (4) SalB+LPS group: cells were treated with 100 ng/mL LPS and SalB (10^–6^ M) for 90 min; (5) LPS+SalB group: cells were pretreated with 100 ng/mL LPS for 30 min, then SalB (10^–6^ M) was added to the cell culture and incubated for further 60 min; (6) Control group: cells were treated with PBS for 90 min. In another set of experiments, cells were incubated with 10 μM PP2, an inhibitor for Src family kinases (Sigma Chemical, St. Louis, Missouri, USA) for 1 h before incubation with LPS for 30 min or 90 min.

### Cellular protein extract preparation and Western blot analysis

Cells were lysed in RIPA (50 mM Tris–HCl pH 7.4, 150 mM sodium chloride, 1% NP40, 0.5% sodium deoxycholate, and 0.1% sodium dodecyl sulfate with protease and phosphates inhibitor cocktail, Amresco, Solon, Ohio, USA) and centrifuged at 19 000 g for 15 min. The concentration of supernatants was determined by MicroBCA (Pierce, Rockford, Illinois, USA). Supernatants (10 μg for caveolin-1 and β-tubulin, 20 μg for Src, VE-cadherin and ZO-1, 40 μg for caveolin-1 (Tyr14), Src (Tyr416) and VE-cadherin (Tyr658)) were separated on 10% SDS-PAGE gels and transferred to polyvinylidene difluoride membranes. The membranes were then incubated overnight at 4°C with primary antibodies against β-tubulin, caveolin-1, Src, caveolin-1 (Tyr14) and Src (Tyr416) (Cell Signaling, Beverly, Massachusetts, USA), VE-cadherin (Tyr658) (abcam, Cambridge, USA), VE-cadherin and ZO-1 (Santa Cruz Biotechnology, Santa Cruz, California, USA). The ECL method was used and developed with Kodak XAR film. The protein amount was estimated by quantifying the intensity of protein bands using Quantity one software (Bio-Rad, California, USA).

### Immunofluorescence staining and quantification

Cells were fixed with 4% paraformaldehyde and permeabilized with 0.25% Triton-X100, then incubated with the primary antibodies against caveolin-1, Src, VE-cadherin and ZO-1. Cells were then washed and incubated for 1 h with Dylight-488 or Dylight-549 conjugated secondary antibodies, and with Alexa Fluor-594-conjugated phalloidin (invitrogen, Carlsbad, California, USA) to stain actin stress fibers. Hoechst 33342 was applied to stain the nuclei. Images were captured using a laser scanning confocal microscope (TCS SP50, Leica, Mannheim, Germany).

Confocal Z-stacks of 10 images collected over a depth of 5 μm were projected as one composite image. Five visual fields were selected from each section for analysis of the protein expression using Image-Pro Plus 6.0 software. The fluorescence intensities of caveolin-1, ZO-1, VE-cadherin, F-actin were measured on projected images, normalized to the density of the cells and expressed as fluorescence intensity per cell. Statistical differences between mean values of rank scores for each group were analyzed by one-way ANOVA.

### Surface plasmon resonance

Carboxymethylated (CM5) sensor chip was docked into the BIAcore 3000 (GE Healthcare, Little Chalfont, Buckinghamshire, UK). Binding activity of human recombinant Src immobilized on the CM5 chip was assayed using Src antibody at an increasing concentration. The capture and recovery of the Src binding proteins was conducted according to the RECOVERY program encoded in the instrument.

The rate constants of association and dissociation were derived at five different injection concentrations of SalB. The binding responses were recorded continuously in response units. The association (Ka) and dissociation (Kd) rate constants, and the equilibrium dissociation constant (KD) were determined by the analysis of the sensorgram curves obtained at different concentrations of SalB by the use of BIA evaluation software version 4.1 software (GE Healthcare, Little Chalfont, Buckinghamshire, UK) and the 1:1 Langmuir binding fitting model. The curve fitting efficiency was evaluated by statistical parameter χ2.

### BioLayer interferometry

BioLayer Interferometry (BLI) is a label-free technology for measuring binding kinetics and affinities of biomolecular interactions. All BLI experiments were performed using an Octet Red instrument (ForteBio Inc., California, USA). For the pre-equilibrium the streptavidin–coated biosensor tip was incubated in PBS (pH 7.4), then biotinylated Src antibody (abcam, Cambridge, USA) was loaded onto the biosensors. SSA biosensors were then loaded with recombinant human Src (Sigma Chemical, St. Louis, MO, USA). After washing the tip in PBS, the association was performed with various doses of SalB. Affinities were determined by fitting the kinetic data to a 1:1 Langmuir binding model utilizing global fitting algorithms to derive Ka, Kd and KD values. Binding affinities were calculated using ForteBio Data Acquisition 7.1 software (ForteBio Inc., California, USA).

### Statistical analysis

The results are expressed as mean±SE. For comparison of >2 conditions a one-way analysis of variance (ANOVA) with Turkey post hoc test or a repeated measures ANOVA with Bonferroni post hoc test were used. A value of P < 0.05 was considered statistically significant.

## Results

### SalB attenuates albumin leakage from rat mesenteric venules induced by LPS

**Fig 1:**
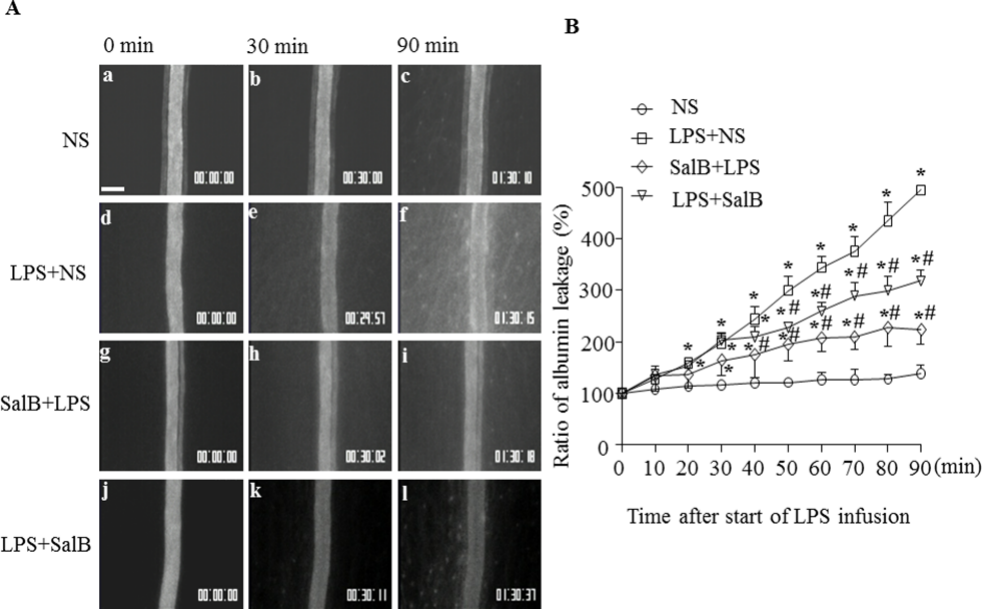
The influence of SalB on albumin leakage from rat mesenteric venules. **A**: Representative images of fluorescence microscopy showing rat mesenteric venules at baseline (0 min), and 30 and 90 min after reagent infusion in different groups. Bar = 50 μm. **B**: Time course of albumin leakage form venules in different groups. Data were expressed as mean±SE of 6 animals. * *p* < 0.05 vs. NS group; # *p* < 0.05 vs. LPS group. For albumin leakage analysis, repeated measures ANOVA with Bonferroni post hoc test were used.

The permeability of post-capillary venules in different groups was assessed by FITC-albumin leakage. The representative images for each group at 0, 30, and 90 min after infusion are presented in Fig 1A, and the time course of albumin leakage in each group depicted in 1B. Obviously, the albumin leakage in normal saline group remained at a negligible level over the 90 min of observation. In contrast, albumin leakage increased impressively with time in LPS group, being significantly different as compared with control starting from 20 min till 90 min. Of notice, the LPS-elicited albumin leakage was attenuated by administration of SalB, and the role of SalB was more prominent in SalB+LPS group than that in LPS+SalB group.

### SalB decreases the number of vesicles in venular endothelial cells in rat mesentery exposed to LPS

**Fig 2:**
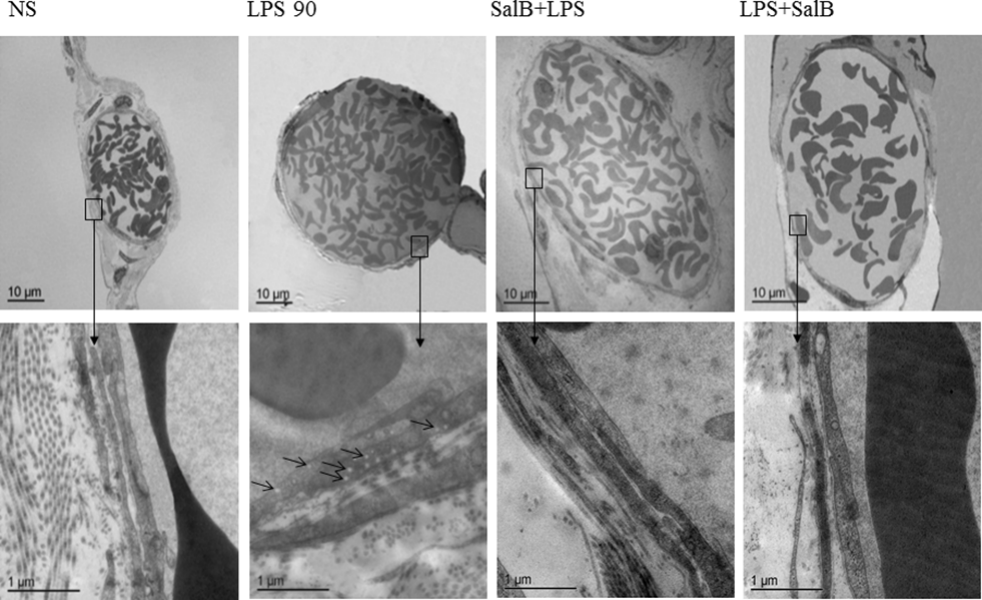
Effect of SalB on the number of caveolae in endothelial cells of rat microvascular venules. Displayed are electron micrograms of rat mesenteric venules in different groups with high magnifications shown below. Arrows indicate caveolae.

Post-capillary venules of rat mesentery were examined by electron microscopy. The representative transmission electron micrographs of venules in rat mesentery are presented in Fig 2. The lumens of venules in rat mesentery infused with normal saline were lined by a layer of endothelial cells with few vesicles in the cytoplasm. LPS infusion led to an apparent alteration in the ultrastructure of the endothelial cells, characterized by plentiful vesicles of different size in the cytoplasm. The LPS-induced increase in the number of vesicles was remarkably abated by infusion with SalB.

### SalB decreases protein level and phosphorylation of caveolin-1 in endothelial cells exposed to LPS

**Fig 3:**
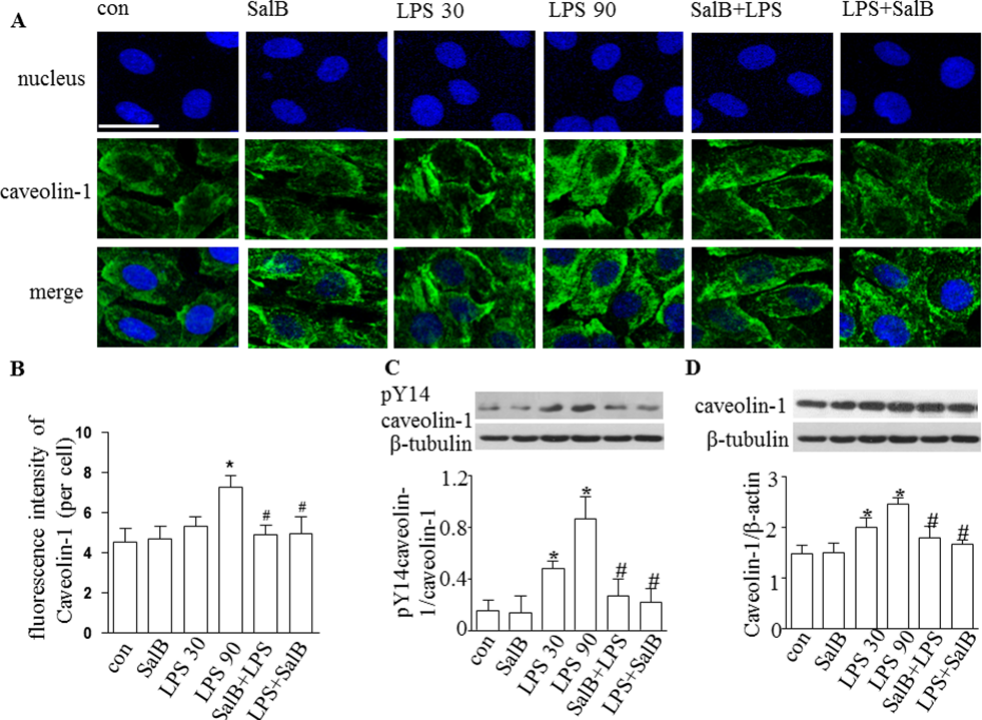
Effect of SalB on caveolin-1 in HUVECs subjected to LPS challenge. **A:** Images of confocal microscopy showing the distribution of caveolin-1 in HUVECs in different groups. Bar = 25 μm. **B:** Quantitative assessment of caveolin-1 in HUVECs. C: Western blot showing the protein level of phosphorylation of caveolin-1 (Tyr14) and the ratio of phosphorylated caveolin-1 (Tyr14) to caveolin-1 in HUVECs in different groups. **D:** Western blot showing the protein level of caveolin-1 and the ratio of caveolin-1/β-tubulin in HUVECs in different groups. Data were expressed as mean±SE of 6 independent experiments. *p <0.05 vs. control group, # *p*<0.05 vs. LPS 90 group. One-way analysis of variance (ANOVA) with Turkey post hoc test was used.

Fig 3A illustrates the distribution of caveolin-1 in HUVECs examined by confocal microscope. Caveolin-1 controls the formation and fission of caveolae from the plasma membrane, which shuttle macromolecules, such as albumin, across the endothelial barrier. Confocal microscope revealed a localization of caveolin-1 in the cytoplasm and plasma membrane of HUVECs, which was increased by LPS treatment for 30 min and 90 min, as compared to control, implying an involvement of caveolae in endothelial hyperpearmeability after LPS. Treatment with SalB attenuated caveolin-1 obviously, indicating that caveolin-1 was implicated in the regulation of vascular permeability by SalB, in accordance with the mean density of caveolin-1 measured in Fig 3B.

Western blot analysis of proteins from HUVECs confirmed the results above, and further demonstrated that caveolin-1 regulated vascular permeability in the present setting through altering both expression and phosphorylation (Fig 3C and 3D).

### SalB attenuates the degradation of ZO-1, the translocation of VE-cadherin and the rearrangement of F-actin in HUVEC induced by LPS

**Fig 4:**
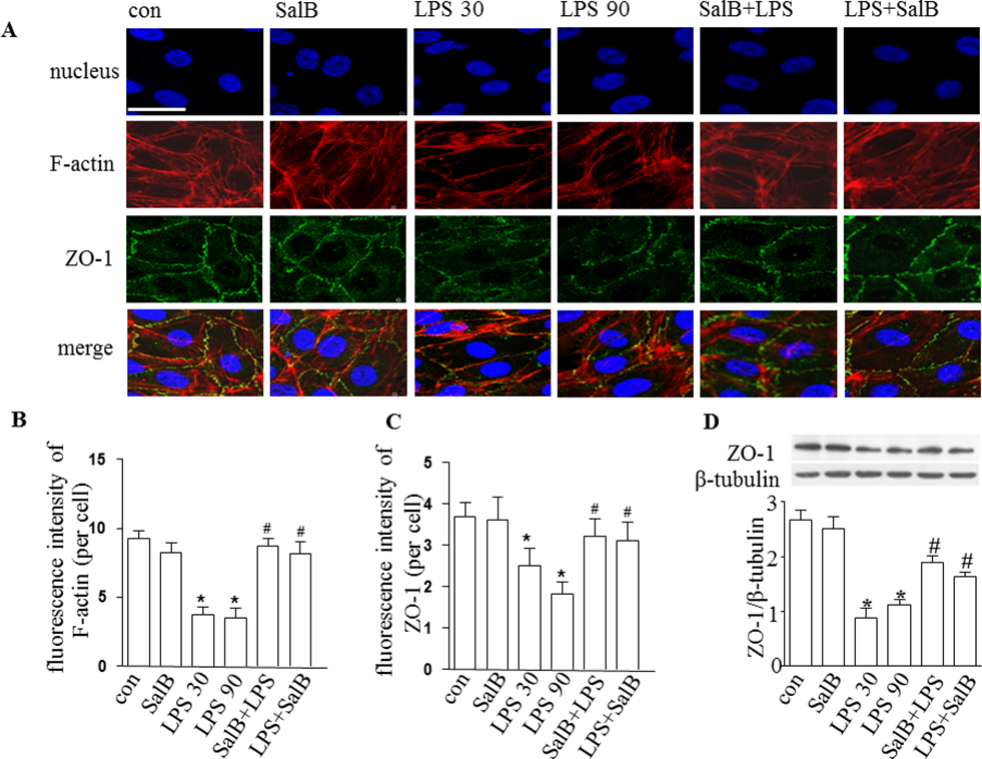
Effect of SalB on the distribution and expression of ZO-1 in HUVECs. **A:** Representative images of confocal microscopy showing of distribution of ZO-1 (green) and F-actin (red) in HUVECs in different groups. Hoechst 33342 was applied to stain nuclei (blue). Bar = 25 μm. **B:** Quantitative assessment of F-actin in HUVECs. **C**: Quantitative assessment of ZO-1 in HUVECs. **D**: Western blot showing protein level of ZO-1 the ratio of ZO-1/β-tubulin in HUVECs in different groups. Data were expressed as mean±SE of 6 independent experiments. * *p* < 0.05 vs. control group; # *p* < 0.05 vs. LPS 30 and LPS 90 groups. One-way analysis of variance (ANOVA) with Turkey post hoc test was used.

**Fig 5:**
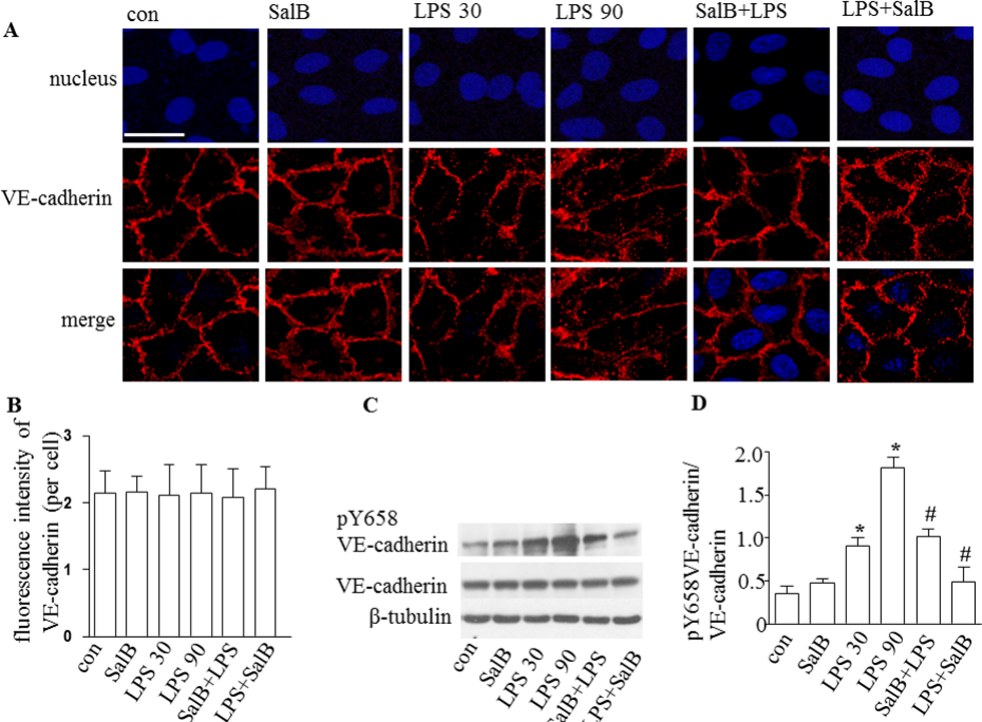
Effect of SalB on the distribution and expression of VE-cadherin in HUVECs. **A**: Representative images of confocal microscopy showing distribution VE-cadherin (red) in HUVECs in different groups. Hoechst 33342 was applied to stain nuclei (blue).Bar = 25 μm. **B:** Quantitative assessment of VE-cadherin in HUVECs. **C**: Western blot showing protein level of VE-cadherin and phosphorylation of VE-cadherin (Tyr658) in HUVECs in different groups. **D**: Quantification of Western blot results for phosphorylation of VE-cadherin (Tyr658) in HUVECs in different groups. Data were expressed as mean±SE of 6 independent experiments. * *p* < 0.05 vs. control group; # *p* < 0.05 vs. LPS 30 and LPS 90 groups. One-way analysis of variance (ANOVA) with Turkey post hoc test was used.

To address the alteration of endothelial cell junctions in response to LPS and SalB, we next examined TJs and AJs in HUVECs by confocal microscopy and Western blot. After immunofluorescence staining, confocal microscopy showed a uniform distribution of ZO-1 and VE-cadherin at the cell periphery and a well arranged F-actin in untreated HUVEC monolayers. LPS exposure induced a range of alterations in the cells including reduction in F-actin and ZO-1 staining and the retraction of the cell mass toward the center (Figs 4A and 5A). Western blot confirmed this presumption, showing that LPS decreased F-actin and ZO-1 protein expression (Fig 4D), while had no effect on VE-cadherin expression but increased its phosphorylation (Tyr658) (Fig 5C and 5D). Of notice, all the LPS-induced changes in junction proteins and F-actin were ameliorated significantly by administration of SalB, suggesting that SalB attenuated rat mesentery microvascular permeability induced by LPS via both interendothelial and transendothelial pathway.

### SalB inhibits phosphorylation of Src (Tyr416) in HUVEC induced by LPS

**Fig 6:**
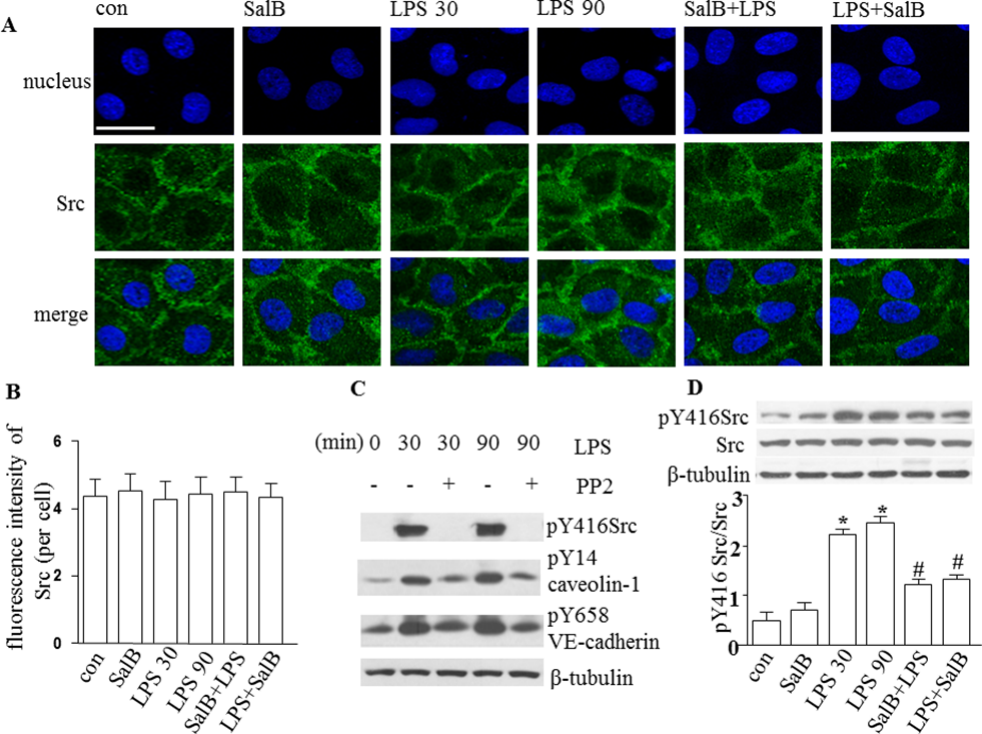
Effect of SalB on Src expression and phosphorylation in HUVECs exposed to LPS. **A**: Representative images of confocal microscopy showing the distribution of Src (green) in HUVECs in different groups. Hoechst 33342 was applied to stain nuclei (blue). Bar = 25 μm. **B:** Quantitative assessment of Src in HUVECs. **C:** Western blot showing the effect of Src inhibitor PP2 (10 umol/L) on phosphorylation of Src, caveolin-1 and VE-cadherin in HUVECs exposed to LPS. **D**: Western blot showing the level of Src and phosphorylated Src (Tyr416) and quantification of the level of phosphorylation of Src (Tyr416) in HUVECs in different groups. Data were expressed as mean±SE of 6 independent experiments. * *p* < 0.05 vs. control group; # *p* < 0.05 vs. LPS 30 and LPS 90 groups. One-way analysis of variance (ANOVA) with Turkey post hoc test was used.

Src has been implicated in upstream signaling pathways that lead to endothelial hyperpermeability. To investigate the role of Src in our setting, we used PP2, the specific inhibitor of Src, to treat HUVECs for 30 min before administration of LPS. Western blot showed that PP2 blocked the phosphorylation of Src completely, and phosphorylation of caveolin-1 and VE-cadherin significantly, after LPS, suggesting Src as the key molecule in regulating the activity of caveolin-1 and VE-cadherin (Fig 6C).

Next, we explored the effect of SalB on Src in HUVECs treated with LPS by using confocal microscopy and Western blot. Confocal microscopy revealed that either LPS or SalB treatment for 90 min had no effect on the distribution of Src in HUVECs (Fig 6A and 6B). Western blot showed that treatment with LPS for 30 and 90 min increased the phosphorylation of Src (Tyr416), which was blunted by SalB significantly (Fig 6D).

The results above indicate that Src plays a key role as the upstream signaling pathway in the action of SalB on microvascular hyperpermeability induced by LPS.

### SalB is able to bind to Src in a dose-dependent manner

**Fig 7:**
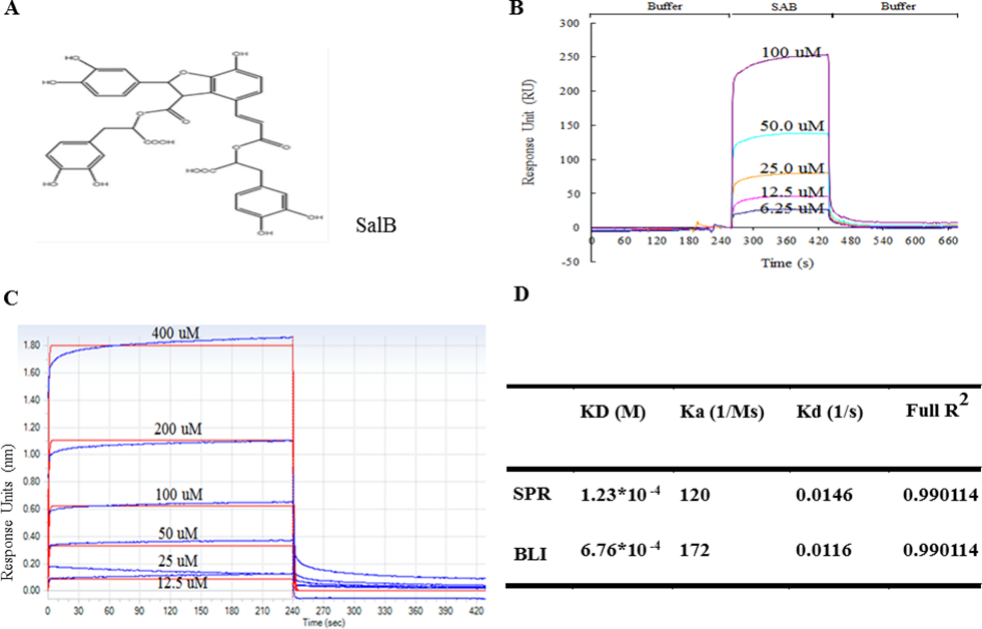
A: The chemical structure of SalB and the kinetic binding of SalB to Src. **B**: Binding assay by SPR. **C**: Binding assay by BLI. Binding assays were performed using a series of 2-fold diluted human Src solution. Association time was set at 10 min and dissociation time was set at 30 min. Affinities were derived by fitting the kinetic data to a 1:1 Langmuir binding model utilizing global fitting algorithms for determination of the association rate (K_a_), dissociation rate (K_d_), and affinity (K_D_) of biomolecule interactions. **D:** K_a_, K_d_ and K_D_ values derived from SPR and BLI. Data were expressed as mean±SE of 6 independent experiments.

In view of the critical role of Src in SalB (the chemical structure is shown in Fig 7A) action, we determined the interaction between Src and SalB by using SPR and BIL. As shown in Fig 7B and 7C, SalB bound to Src in a dose-dependent manner. Fig 7D presented the K_a_, K_d_ and K_D_ obtained by SPR and BIL, indicating that SalB can directly bind to Src.

## Discussion

The present study demonstrated the potential of SalB to ameliorate the LPS-induced microvascular hyperpermeability, as shown by the decrease in albumin leakage from rat mesenteric venules. As to the mechanism therefore, we observed an ability of SalB to reduce the caveola number in rat mesenteric venular endothelial cells after LPS challenge, and decrease the expression and phosphorylation of caveolin-1, the redistribution and phosphorylation of VE-cadherin, and the degradation of ZO-1 in HVUECs exposed to LPS. Furthermore, SalB was able to bind to Src and inhibit its phosphorylation.

Previous researches have shown the multiple potentials of SalB, including anti-oxidative and anti-inflammatory actions in different animal models, and suggest the likelihood of SalB as a remedy to improve microvascular hyperpermeability [18, 23, 24]. In the present study, we provided direct evidence demonstrating that SalB was able to ameliorate LPS-provoked albumin leakage from rat mesenteric venules. It is now well accepted that albumin leakage due to increased vascular endothelial permeability contributes to the pathogenesis of effective circulating blood volume deficiency or even hypovolemic shock [25]. A number of medicines, such as albumin and hydroxyethyl starch, have been applied to treat circulating blood volume deficiency in clinic, but they are often ineffective if administrated when microvascular permeability has increased and plasma macromolecules have leaked [26]. In HUVEC of the present study, SalB had almost same effects in both SalB+LPS and LPS+SalB, or relatively more effects in LPS+SalB, whereas in vivo study, there was less effects in LPS+SalB. The reason for this difference is unclear. One possibility is that in vitro HUVEC are exposed to a constant concentration of SalB while in vivo the vascular endothelial cells are exposed to SalB at a concentration that is changing with time due to blood circulation and drug metabolism. Importantly, nevertheless, both in vivo and in vitro study showed that SalB is effective, irrespective of SalB administration simultaneous with LPS or 30 min after LPS infusion. This result points to the possibility of application of SalB in clinic as an alternative for treatment of microvascular hyperpermeability-related diseases. Nevertheless, clinical translation of SalB for this purpose needs more studies.

Microvascular permeability is depending not only on the endothelium integrity but also the property of extracellular matrix proteins. As a result, a complicated mechanism must exist to maintain the normal basal microvascular permeability and regulate it whenever necessary. In the present study, FITC-conjugated albumin was used as a tracer, which, in conjunction with intravital microscopy, enabled a real-time assessment of the venular permeability. By means of this technique, a negligible albumin leakage was found at basal state, while a rapid increase in venular permeability was detected in response to LPS exposure, which started from 10 min after LPS infusion, the earliest time point of observation, and persisted increase with time over the period of examination. This result indicates that the venular endothelium permeability was regulated by a highly responsive mechanism in the present setting. Our in vivo experiment revealed a prominent increase in the number of small intracellular vesicles in vascular endothelial cells after LPS challenge, which was attenuated by SalB treatment, suggesting contribution of transcellular pathway to this mechanism. The nature of these small intracellular vesicles we observed is to be identified further. So far, two types of small intracellular vesicles are reported in vascular endothelial cells, caveolae and vesiculo-vacuolar organelles (VVOs), which, though, are indistinguishable morphologically by conventional transmission electron microscopy, but differ in composition and, possibly, the opening machinery [27]. Nevertheless, our in vitro experiments by immunofluorescence staining and Western blot showed that caveolin-1 expression and phosphorylation in HUVECs changed in response to LPS and SalB treatment in a manner similar to small intracellular vesicles in venular endothelial cells in vivo, suggesting caveolae as the major, if not only, contributor to the transcellular pathway in the present case. Caveolin-1, an integral membrane protein (20–22 kDa), is a specific marker and the primary structural component of caveolae, and the level of cavelin-1 expression correlates with the number of caveolae [28]. Phosphorylation of caveolin-1 on tyrosine residue 14 initiates plasmalemmal vesicle fission and facilitates transendothelial vesicular transport [15]. Furthermore, the experiments carried out in HUVECs showed that LPS exposure provoked a significant decrease in the expression of ZO-1 and an increase in phosphorylation of VE-cadherin, as well as a change in the distribution of F-actin and VE-cadherin, indicative of disruption of tight junctions and adherins junctions, which is consistent with the results from others [29]. Importantly, the LPS-elicited alteration in endothelial cell junction proteins and cell skeleton was restored by treatment with SalB, indicating that the protection of SalB against microvascular hyperpermeability after LPS involves regulation of intercellular pathway as well. The potential of SalB to regulate both para- and trans-endothelial cell pathways to modulate endothelium permeability implies that it presumably acts at a target that controls the two routes. We speculated that this target is Src.

Src family protein tyrosine kinases are nonreceptor, cytoplasmic protein kineses with diverse molecules as their substrates. The role of Src signaling in regulation of microvascular permeability has received increasing attention. For paracellular permeability, activated Src is known to be able to disrupt the integrity of intercellular junctions by phosphorylation of myosin light chain kinase and adherens junction protein VE-cadheren [15]. Src activation has been reported to signal the LPS-reduced ZO-1 expression [30]. For transcellular permeability, Src activation is accepted as an initiator for caveolin-1 phosphorylation to trigger caveolae fission from plasma membrane and transcellular transport [15]. On the other hand, in spite of the consensus on the critical role of Src in regulation of microvascular permeability, a strategy for the treatment of Src-mediated vascular leakage is not yet available [15]. The results of the present study showed that SalB may potentially be used as such a strategy. The notion that SalB attenuates LPS-elicited microvascular hyperpermeability via inhibition of Src was further verified by the findings that SalB indeed inhibited Src activation, and that PP2, a specific inhibitor of Src, displayed a similar effect as SalB did on inhibition of phosphorylation of caveolin-1 and VE-cadherin. If Src serves as the target for SalB action, a direct interaction between the two molecules should occur. To test this speculation, we applied two techniques, surface plasmon resonance and biolayer interferometry, in the present study, the results from both of which indicate that SalB binds to Src with a high affinity. The mechanism for this binding is suggested by a recent report showing that SalB may bind to SH_2_ domains of the Src-family kinases and act as an inhibitor of the protein-protein interactions mediated by the Src-family kinases [31]. Nonetheless, the rationale for SalB to inhibit Src activity needs to be clarified by further study, and the possibility that SalB binds to other SH_2_ domain-containing target to exert action needs to be excluded.

In conclusion, SalB is able to ameliorate albumin leakage from rat mesenteric venules through regulating both paracelluar and transcelluar pathways by inhibition of Src activation. These findings suggest SalB as an option to treat microvascular barrier dysfunction-related diseases, such as endotoxmia, adult respiratory distress syndrome and shock, and provide insight for understanding the mechanism thereof.
